# Multiplex gene analysis reveals T-cell and antibody-mediated rejection-specific upregulation of complement in renal transplants

**DOI:** 10.1038/s41598-021-94954-3

**Published:** 2021-07-29

**Authors:** Eva Vonbrunn, Tajana Ries, Stefan Söllner, Janina Müller-Deile, Maike Büttner-Herold, Kerstin Amann, Christoph Daniel

**Affiliations:** 1 Department of Nephropathology, Friedrich-Alexander-University (FAU) Erlangen-Nuernberg, Krankenhausstr. 8-10, 91054 Erlangen, Germany; 2 Department of Nephrology and Hypertension, Friedrich-Alexander-University (FAU) Erlangen-Nuernberg, Erlangen, Germany

**Keywords:** Immunology, Complement cascade, Transplant immunology

## Abstract

In renal transplantation, complement is involved in ischemia reperfusion injury, graft rejection and dysfunction. However, it is still unclear how induction of complement and its activation are initiated. Using allograft biopsies of a well-characterized cohort of 28 renal transplant patients with no rejection (Ctrl), delayed graft function (DGF), acute T-cell-mediated (TCMR) or antibody-mediated rejection (ABMR) we analyzed differences in complement reaction. For that mRNA was isolated from FFPE sections, quantified with a multiplex gene expression panel and correlated with transplant conditions and follow-up of patients. Additionally, inflammatory cells were quantified by multiplex immunohistochemistry. In allograft biopsies with TCMR and ABMR gene expression of C1QB was 2-4 fold elevated compared to Ctrl. In TCMR biopsies, mRNA counts of several complement-related genes including C1S, C3, CFB and complement regulators CFH, CR1 and SERPING1 were significantly increased compared to Ctrl. Interestingly, expression levels of about 75% of the analyzed complement related genes correlated with cold ischemia time (CIT) and markers of inflammation. In conclusion, this study suggest an important role of complement in transplant pathology which seems to be at least in part triggered by CIT. Multiplex mRNA analysis might be a useful method to refine diagnosis and explore new pathways involved in rejection.

## Introduction

Given the shortage of kidneys for transplantation, it is essential to preserve transplant function as good as possible. The complement system is known to be involved in the pathogenesis of various renal diseases^1^ and in transplant injury^2^. Activation of the complement system is of central importance for the immune response in solid organ transplants, not only in the context of ischemia reperfusion damage^3^, but also by enhancing T-cell and B-cell mediated immunity^4^. A growing understanding of the role of the complement system and its pharmacological modulation are therefore promising approaches to reduce transplant-damage and thereby prolong the grafts survival^2^. The complement system consists of more than 40 proteins, comprising membrane bound and soluble molecules, including receptors and regulatory proteins^5^. Three different complement pathways exist—the classical, lectin and alternative^6^. The three activation pathways converge at complement component 3, which is enzymatically cleaved by the C3 convertase to the bioactive fragments C3a and C3b^7^. Subsequently the terminal complement pathway is activated by cleavage of C5 through the C5 convertase, finally resulting in formation of the membrane attack complex (MAC) C5b-9, which forms pores in the target membrane^8^. The MAC is a critical effector in mediating complement-induced post-ischemic injury in the kidney^9^.

Tissue deposition of C4d is a well-established marker for antibody-mediated rejection (ABMR) and associated with reduced long-term graft survival^10^. To discover distinctive features of graft rejection we compared kidney transplant biopsies of patients with delayed graft function (DGF), acute T-cell-mediated rejection (TCMR) and ABMR with a control group without rejection (Ctrl) regarding their complement expression. Previous mRNA expression studies in kidney transplants were performed using microarrays (cryo or RNAlater-fixed material) but required a 2nd biopsy taken for this purpose only. These studies have already shown that the innate immune system plays a significant role in graft rejection, but have never focused directly on the complement system^11,12^. In our study transcript analysis was conducted with the NanoString nCounter® FLEX Analysis System using the Human Organ Transplant Panel, which was created through a collaboration between NanoString and the Banff Foundation for Allograft Pathology^13^. This high through-put gene expression platform has the ability of analyzing up to 800 genes per sample and works on formalin-fixed paraffin-embedded (FFPE) tissue^14,15^. This allows expression analysis and histology on the same biopsy and thus direct correlation of the findings^16^. In addition, we correlated our results with relevant clinical data like cold and warm ischemia time and recipient and donor data like age, body mass index (BMI), serum creatinine, renal inflammation and living or deceased donor state.

## Results

### Local upregulation of complement components in ABMR and TCMR

**Figure 1:**
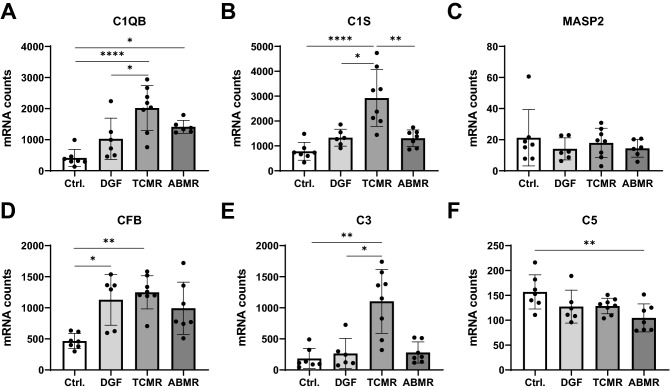
Expression analysis of complement initiator molecules and factors. Number of mRNA molecules coding for the initiator molecules C1QB (**A**, ABMR n = 6), C1S (**B**), MASP2 (**C**, ABMR n = 6) and CFB (**D**), and the complement factors C3 (**E**) and C5 (**F**) in follow-up biopsies of renal transplants with no rejection or dysfunction (Ctrl), delayed graft function (DGF), T-cell mediated rejection (TCMR) or antibody mediated rejection (ABMR); (Statistical analysis: **A**, **B**, **F**: ANOVA with Tukey’s; **C**–**E**: Kruskal–Wallis with Dunn’s; *p < 0.05; **p < 0.01; ***p < 0.001; ****p < 0.0001).

Gene expression of 28 follow-up biopsies of renal transplants with either DGF, TCMR, ABMR or no rejection/dysfunction (Ctrl) were compared regarding expression of complement components. Expression of several complement initiator molecules, proteases and factors was upregulated in biopsies of renal transplants with TCMR and ABMR. The mRNA of C1QB, the initiator molecule of the classical pathway, was significantly upregulated in TCMR (fourfold) and ABMR biopsies (threefold), compared to Ctrl (Fig. 1A). In addition, the corresponding protease C1S was markedly higher expressed in TCMR biopsies than in the three other groups, while mean C1S expression in DGF and ABMR was less than twofold increased compared to Ctrl (n.s., Fig. 1B). Furthermore, the lectin pathway protease MASP2 was expressed only on a very low level and did not differ between the four groups (Fig. 1C). In contrast, the alternative pathway protease CFB showed significantly increased expression in TCMR and DGF samples (more than twofold) compared to Ctrl (Fig. 1D). C3, as central component of all complement pathways, was only significantly upregulated in TCMR biopsies compared to Ctrl and DGF (Fig. 1E). The terminal complement pathway factor C5 was expressed on a low level with significantly lower expression in ABMR versus Ctrl biopsies (Fig. 1F).

### Regulation of complement receptors and inhibitors in ABMR and TCMR

**Figure 2:**
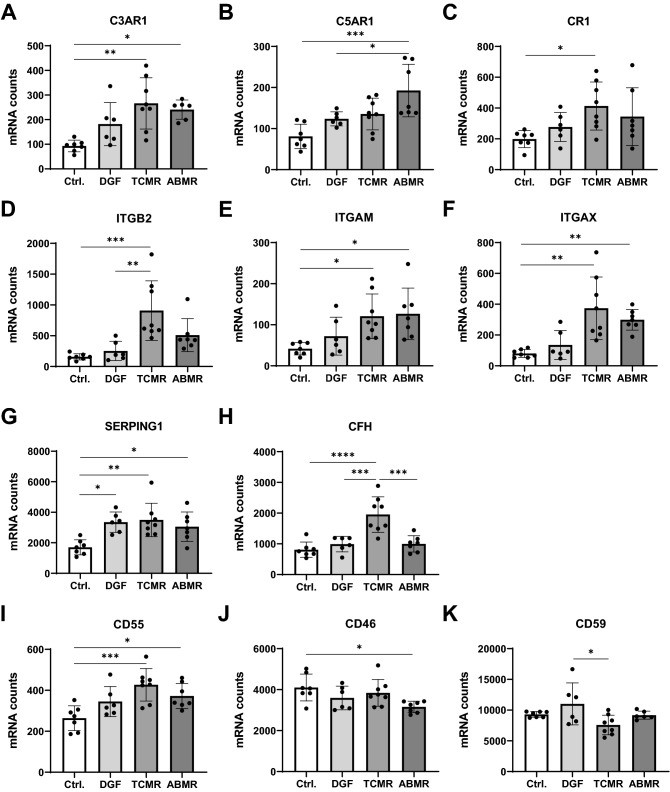
Expression analysis of complement receptors and complement inhibitors. Number of mRNA molecules coding for the complement receptors C3AR1 (**A**, ABMR = 6), C5AR1 (**B**), CR1 (**C**), ITGB2 (**D**), ITGAM (**E**) and ITGAX (**F**) and complement inhibitors SERPING1 (**G**), CFH (**H**) CD55 (**I**, ABMR = 6), CD46 (**J**) and CD59 (**K**) in follow-up biopsies of renal transplants with no rejection or dysfunction (Ctrl), delayed graft function (DGF), T-cell mediated rejection (TCMR) or antibody mediated rejection (ABMR); (Statistical analysis: **B**, **C**, **E**, **G**–**K**: ANOVA with Tukey’s; **A**, **D**, **F**: Kruskal–Wallis with Dunn’s; *p < 0.05; **p < 0.01; ***p < 0.001; ****p < 0.0001).

In the same kidney biopsies, expression of regulatory components of the complement system like receptors and inhibitors was also analyzed. Mean expression of anaphylatoxin receptors C3AR1 and C5AR1 was increased in all 3 groups compared to Ctrl. However, these differences were only statistically significant in the ABMR group, while in the TCMR group only C3AR1 was significantly increased (Fig. 2A,B) compared to Ctrl. Complement receptor 1 (CR1) was significantly enhanced in TCMR compared to Ctrl (Fig. 2C), which was also true for ITGB2 (CD18), a protein that combines with ITGAM and ITGAX to form CR3 and CR4 (Fig. 2D). Both ITGAM and ITGAX were also significantly upregulated in TCMR and ABMR compared to Ctrl (Fig. 2E,F). Mean gene expression values in the DGF group were generally slightly increased compared to Ctrl (n.s., Fig. 2). However, C5AR1 was expressed significantly fainter in DGF than in ABMR (Fig. 2B) and ITGB2 was expressed significantly less than in TCMR (Fig. 2D,F). Soluble and membrane bound inhibitors control complement activation in vivo*,* to prevent an undesired or exaggerated complement reaction. C1 inhibitor SERPING1, that regulates the classical and the lectin pathway, was about 2-fold increased in DGF and TCMR biopsies and 1.7-fold in ABMR compared to Ctrl (Fig. 2G). CFH, as inhibitor of the alternative pathway, was more than twofold upregulated in kidneys with TCMR compared to other groups (Fig. 2H). The mRNA levels of the complement decay-accelerating factor CD55, that binds C4b and C3b preventing the formation of the protein complexes C4b2a and C3bBb was slightly but significantly increased in TCMR and ABMR biopsies compared to Ctrl (Fig. 2I). In contrast, the co-factor CD46 that is involved in cleavage of C3b and C4b showed a significantly lower expression in ABMR versus Ctrl (Fig. 2J). As part of the terminal pathway CD59, that can act as MAC-inhibitor, was significantly lower expressed in TCMR versus DGF (Fig. 2K).

### Complement expression correlates with cold ischemia time (CIT)

**Figure 3:**
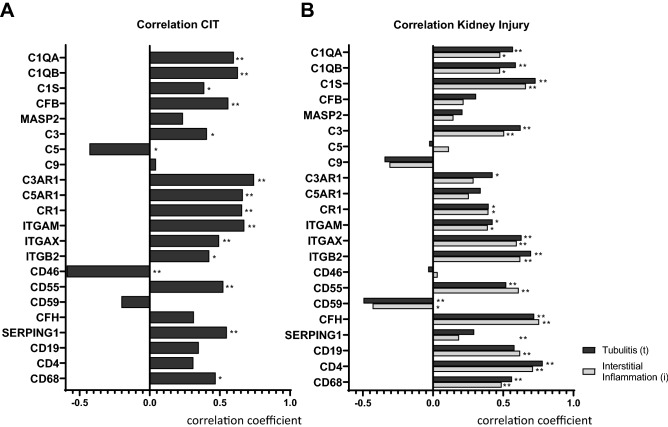
Correlation analysis of complement gene expression with CIT and kidney inflammation in transplant biopsies. Correlation of complement system-associated gene RNA molecule numbers with cold ischemia time during transplantation (**A**) and severity of tubulitis and interstitial inflammation (**B**) in follow-up biopsies; (Statistical analysis: Spearman correlation test, *p < 0.05; **p < 0.01); n = 28.

Correlation of the NanoString complement expression data with transplantation relevant clinical parameters at biopsy revealed the following results: Serum creatinine at biopsy correlated positively with expression of some complement factors being involved in the classical pathway, including C1QA and C1QB (both r = 0.422*), the anaphylatoxin receptors C3AR1 (r = 0.497*) and C5AR1 (r = 0.583*) and the alternative pathway activator CFB (r = 0.516*) as well as the complement inhibitors CD55 (r = 0.445*) and SERPING1 (r = 0.658**) (Suppl. Table 2). Interestingly, CIT correlated with almost all complement factors and also showed a stronger correlation and often a higher significance level than observed for serum creatinine (Fig. 3A). Early genes of the classical pathway like C1QA, C1QB und C1S, just as the central complement factor C3 and its receptor C3AR1, correlated significantly and positively with CIT. Surprisingly, the terminal pathway factor C5 showed a significant negative correlation, while its corresponding receptor C5AR1 was positively associated with CIT (Fig. 3A). In contrast, C9 and the T- and B-cell surface molecules CD4 and CD19 did not correlate with CIT (Fig. 3A). Complement system inhibitors showed variable results: while CD46 and CD59 expression correlated negatively with CIT, CD55 correlated positively. In contrast to lymphocytes, CD68 as a marker of macrophages, showed a significant positive correlation to CIT. The alternative pathway protease CFB also correlated positively with CIT, while its inhibitor CFH did not (Fig. 3A). Expression of complement receptor CR1 in transplant biopsies was significantly associated with CIT as well as the components of complement receptors 3 and 4: ITGB2, ITGAM and ITGAX. Furthermore, the expression of the C1 inhibitor gene SERPING1 positively correlated with CIT, while expression of lectin pathway protease MASP2 that was found only at background levels showed no correlation (Fig. 3A). No correlation of any complement factor was found for warm ischemia time, donor and recipient age and BMI (data not shown).

### Complement expression correlates with kidney inflammation

Since the complement system is closely linked to the adaptive immune system, the next step was to correlate the expression of complement factors with the severity of tubulitis and interstitial inflammation in renal graft biopsies according to Banff classification. The severity of both, tubulitis and interstitial inflammation in transplant biopsies, i.e. the t- and i-scores, significantly correlated with a variety of complement-associated genes e.g. C1QA, C1QB, C1S as well as C3 and C3AR1 (Fig. 3B). In contrast, expression of terminal complement genes like C5, C5AR1 and C9 showed no significant association with tubulitis and interstitial inflammation (Fig. 3B). As expected, correlation with tubulitis and interstitial inflammation was highly significant for CD19, CD4 and CD68 positive immune cells (Fig. 3B). Inhibitory molecules again interacted variably. While CD46 expression was not linked to inflammation in kidney grafts, CD55 was significantly and positively and CD59 negatively correlated (Fig. 3B). CFH expression correlated with high significance with graft inflammation, whereas CFB expression did not. Moreover, complement receptor genes CR1, ITGAM, ITGAX and ITGB2 showed a significant link between expression and kidney inflammation (Fig. 3B). No correlation could be detected for the expression of the lectin pathway protease MASP2 and the C1 inhibitor SERPINE1 (Fig. 3B).

### Markers of macrophages, T- and B-cell were upregulated in grafts with ABMR and TCMR and correlated with expression of complement components

**Figure 4:**
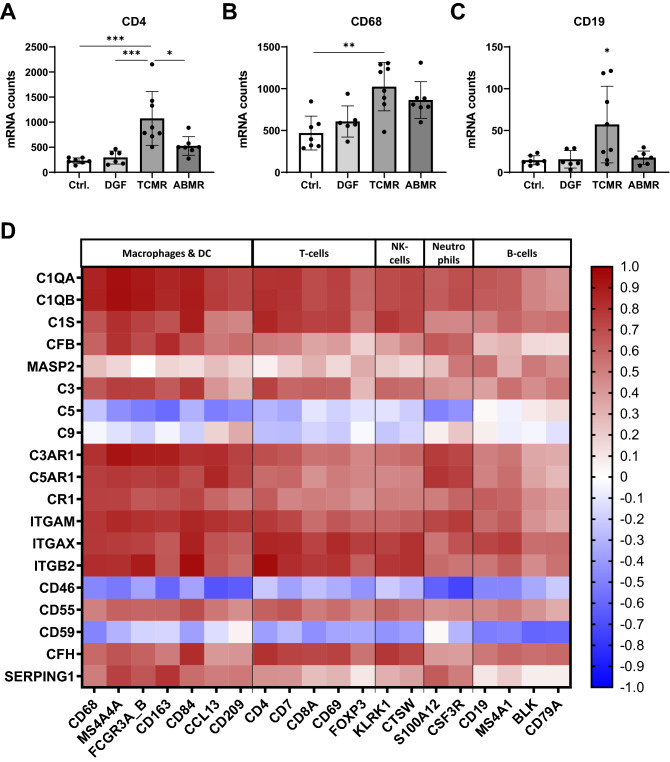
Expression analysis of immune cells. Number of mRNA molecules coding for T-lymphocyte (**A**), macrophage (**B**) and B-lymphocyte (**C**, ABMR n = 6) markers in follow-up biopsies of renal transplants with no rejection (Ctrl), delayed graft function (DGF), T-cell mediated rejection (TCMR) or antibody mediated rejection (ABMR); (Statistical analysis: **A**, **C**: ANOVA with Tukey’s; **B**: Kruskal–Wallis with Dunn’s;*p < 0.05; **p < 0.01; ***p < 0.001). (**D**) Heatmap showing correlation of complement gene expression with the expression of genes specific for macrophages, T-cells, natural killer cells, neutrophils and B-cells; n = 28.

Expression analysis were also performed for CD4-positive T-helper-cells, CD68-positive macrophages and CD19-positive B-cells. As expected, expression of T- and B- cell markers was significantly up-regulated up to 4-times in TCMR compared to ABMR, DGF and the control group (Fig. 4A,C). CD68 mRNA levels was significant increased up to 2-fold in TCMR compared to Ctrl (Fig. 4B). Furthermore, we correlated expression of complement components with markers specific for macrophages, T-cell subsets, B-cells, NK-cells and neutrophils, as shown in a heat map (Fig. 4D). The strongest positive correlations between C1Q subunits, C3 and complement receptor expression were observed with macrophage markers, but correlations with T-cell, NK-cell and neutrophil markers were also strong (Fig. 4D). The weakest but again significant correlation was observed for B-cells and FOXP3, a marker of Treg-cells (Fig. 4D). Significant negative correlation was seen of complement inhibitory receptors CD46 and CD59 with most of the analyzed immune cell markers (Fig. 4D, blue color).

**Figure 5:**
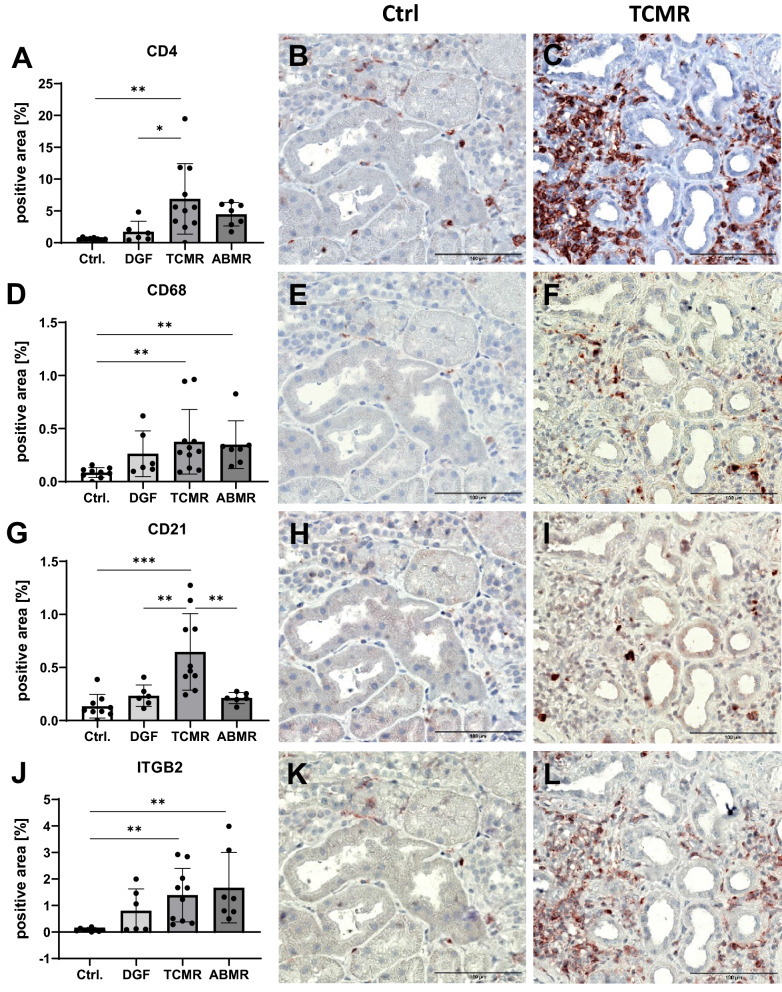
Deposition of immune cells in transplant biopsies. Percentage of positive stained area in follow-up biopsies of renal transplants with no rejection or dysfunction (Ctrl, n = 7), delayed graft function (DGF, n = 6), T-cell mediated rejection (TCMR, n = 8) or antibody mediated rejection (ABMR, n = 7) after immunostaining for CD4 (**A**), CD68 (**D**), CD21 (**G**) and ITGB2 (**J**); (Statistical analysis: **A**, **G**: ANOVA with Tukey’s; **D**, **J**: Kruskal–Wallis with Dunn’s;**p < 0.01; ***p < 0.001; ****p < 0.0001). Examples of biopsies with no rejection or dysfunction (Ctrl) and T-cell mediated rejection (TCMR) after immunostaining for CD4 (**B**, **C**), CD68 (**E**, **F**), CD21 (**H**, **I**) and ITGB2 (**K**, **L**).

In addition, we performed multiplex immunohistochemistry to analyze immune cells in the kidney grafts. The percentage of area stained positive for CD4, CD68 and CD21, confirmed the results of gene expression analysis (Fig. 5). In detail, the amount of CD4-positive T-cells was significantly higher in TCMR compared to DGF or Ctrl (Fig. 5A–C). CD68-positive macrophages were significantly more prevalent in grafts with TCMR and ABMR compared to Ctrl (Fig. 5D–F). Furthermore, anti-CD21 staining for follicular dendritic cells (FDCs) and a subset of B- and T-cells showed a significantly increased number of positive cells in TCMR compared to the other groups (Fig. 5G–I). Since integrin beta 2 (ITGB2) forms the complement receptors CR3 and CR4 together with ITGAM (=CD11b) or ITGAX (=CD11c), respectively, this staining marked macrophages and dendritic cells, that were significantly increased in TCMR and ABMR compared to controls (Fig. 5J–L).

### Principal component analysis

**Figure 6:**
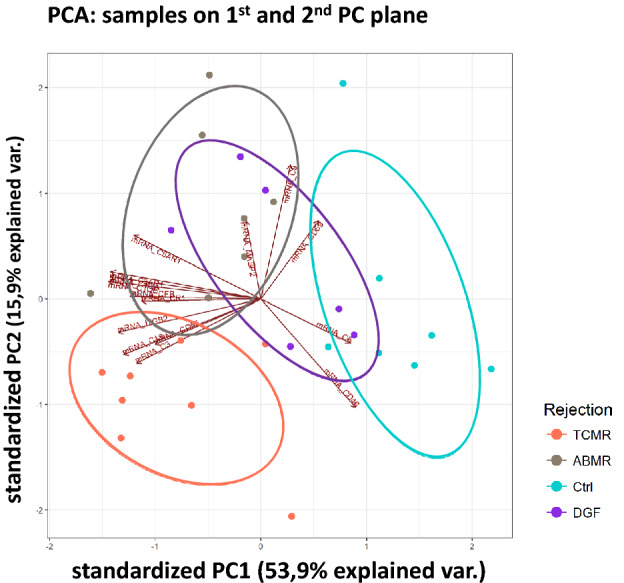
Principal component analysis. Biplot for the expression data of 15 selected complement genes clustered in expression profiles for follow-up biopsies of renal transplants with no rejection or dysfunction (Ctrl, n = 7), delayed graft function (DGF, n = 6), T-cell mediated rejection (TCMR, n = 8) or antibody mediated rejection (ABMR, n = 7).

Finally, we performed principal component analysis using gene expression data of complement related genes (Fig. 6). Using principal component 1 (PC1) and principal component 2 (PC2) in the analysis, TCMR, ABMR and Ctrl can be well differentiated from each other, while DGF samples overlap with Ctrl and ABMR (Fig. 6).

## Discussion

Transplant-related complications such as delayed graft function (DGF), T-cell (TCMR) or antibody-mediated rejection (ABMR) limit graft survival. Factors that trigger these processes are not yet fully understood. The complement system has an important role in kidney damage after transplantation, i.e. complement activation is involved in renal ischemia/reperfusion injury^17–19^, rejection^20–26^ and DGF^27–29^. Therefore, in this study we aimed to further elucidate the involvement of complement by transcriptional analysis of intragraft expression of complement components and inflammatory markers using Nanostring multiplex mRNA analysis in carefully selected renal graft biopsies from patients with well-defined DGF, TCMR and ABMR compared to protocol biopsies with stable graft function. To our knowledge, this is the first study using the multiplex transplant mRNA panel that was designed by the BANFF committee for molecular characterization and diagnostics in solid organ transplantation^13^. In contrast to earlier studies with the need of an additional biopsy for this purpose only or a smaller or pathway specific selection of analyzed transcripts^11,30–32^, we used the new transplant panel investigating 770 transcripts. Hereby, we could confirm data from earlier studies^11^, e.g. high expression of CXCL11 in biopsies with rejection (data not shown). In the present study we specifically focused on the expression of complement components and markers for immune cells. Although it was not the aim of the study to establish the expression of complement factors as discriminators for the different forms of rejection, we could clearly distinguish TCMR and ABMR from stable controls using principal component analysis and also TCMR and ABMR seem to be distinguishable from each other. Only the DGF group showed overlap with Ctrl and ABMR. Therefore, the expression pattern of complement components seems to be specific for ABMR and TCMR. The complement system can be activated by 3 different pathways, the classical, alternative and lectin way. In our study, expression of complement activators that belong to two pathways were increased in rejection. C1QB, which belongs to the classic pathway, was significantly higher expressed in TCMR and ABMR compared to grafts with stable kidney function, arguing for local expression as consequence of the disease process. This local C1q expression is thought to be mediated by interstitial cells and leukocytes (especially macrophages)^33^, which is supported by the extremely strong association of C1q expression with macrophage influx. Clinical trials phase I/II using a C1-esterase inhibitor to block the classical pathway reported less ischemia/reperfusion injury, less rejection events and significant reduction in the need of dialysis and improvement in long-term allograft function in renal transplant patients^34,35^. Although these studies were small, they might indicate a critical involvement of the classical and lectin pathway in renal transplant pathology. Furthermore, mean intragraft expression of the alternative pathway activator complement factor B (CFB), was increased in all groups compared to stable grafts and reached significance in DGF and TCMR. In a rat brain death model blocking of CFB before induction of brain death resulted in preserved renal function and reduced renal damage and inflammation^36^. In another experimental study, Balb/c kidneys transplanted in CFB deficient C57bl6 mice experienced reduced ischemia/reperfusion injury and diminished TCMR^37^. In contrast, MASP-2, involved in activation of the lectin pathway and mediation of ischemia/reperfusion injury^38,39^, was virtually not expressed in our biopsies but not excluding a role of the lectin pathway in transplant pathology. Interestingly, intrarenal C3 was also increased in biopsies with TCMR compared to DGF and stable transplants. In contrast, C5 and C9 expression was very low and barely regulated in transplanted kidneys, confirming that both factors are not predominantly expressed in the kidney but in the liver^40^. All three activation pathways finally lead to the formation of the membrane attack complex (MAC), which lyses bacteria and damages cells by forming pores. In addition to the destruction of cells and invading microorganisms, the complement system also has an immunoregulatory function. The cleavage products C3a and C5a act as anaphylatoxins and bind to their receptors C3aR1 and C5aR1 that are involved in chemotaxis and activation of immune cells and thus promote ischemia/reperfusion injury^41^. C5AR1 deficiency prolongs renal allograft survival in experimental models^42^. In ABMR we found both anaphylatoxin receptors C3AR1 and C5AR1 significantly increased, while in TCMR only C3AR1 expression was significantly higher compared to controls. Since we observed a parallel increase in inflammatory cells in TCMR and ABMR, we assume that the increased expression of complement receptors is rather not due to increased transcription in kidney cells but rather to increased intrarenal inflammatory cells carrying these receptors. This is also true for the complement receptors CR1, CR3, and CR4. Thus, the increased expression of these receptors indicates that complement is involved in rejections. The complement cascade is tightly regulated at different levels to prevent unintended tissue damage. Soluble complement inhibitors such as factor H, factor I and SERPING1, but also inhibitory complement receptors like CD46, CD55 and CD59 prevent a self-harming complement reaction. In patients with a lower intragraft CD55 expression in zero-time biopsies, a significantly faster progression of ABMR was observed 2 years later^43^ and thus CD55 was suggested as a prognostic marker of allograft survival^44^. In contrast, in our study in follow-up biopsies mean expression of CD55 was significantly increased in TCMR and ABMR. However, the expression of inhibitory receptors CD46 and CD59 in our study was only slightly down-regulated and presumably has only a minor influence on the sequence of the complement reaction. However, CD46 and CD59 promotor polymorphism in kidney donors was associated with transplant outcome^45^ and in a rat model of renal transplantation blocking of CD59 resulted in reduced survival compared to non-treated controls^46^. Next, we searched for conditions causing an increased expression of complement components. Nearly all (16/19) complement components analyzed by the multiplex panel significantly correlated positively with CIT. In a retrospective study including a cohort of more than 60.000 patients, CIT has been identified as an important risk factor for acute transplant rejection^47^. Similarly, in another study CIT was identified as a risk factor of DGF^48^. In contrast, Damman et al. showed that in donor kidneys from brain death donors, C3 was already significantly increased at harvest, i.e. before the cold ischemia period, compared to kidneys from living donors^49^. When testing donor origin as a potential confounder, only 4 genes (C1QB, C3AR1, C5AR1 and CD55) correlated significantly with living/deceased donor state. Furthermore, omitting all results derived from living donors from the statistical analyses only changed the level of significance for most genes but not the observed difference per se. Significant differences between groups disappeared for only very few genes including C5, CD59, CR1 and ITGAM, while for others including C1s, C3, CFH and CD46 differences between groups were strengthened when only deceased donors were analyzed (data not shown). However, in our study with exception of the DGF group the time-point of biopsy sampling and the date of transplantation were far apart (sometimes > 2 years). Therefore, we would assume that CIT has an indirect influence on the expression of the complement components maybe by early damage of the graft. The expression of complement components also strongly correlated with tubulitis and interstitial inflammation. The strongest correlation of complement expression was detected with macrophage and dendritic cell markers, but also at high level with T-cell, NK-cell and neutrophil markers was observed. We can only speculate which cells expressed the complement components, as these can be produced by renal cells as well as by the inflammatory cells themselves^50^. Strong correlation of C1Q and C3 expression with T-cell activation marker CD69 and much lower correlation with FOXP3, a marker of regulatory T-cells, suggest that complement is involved in immunostimulatory processes in renal grafts. Local complement expression in the kidney occurs in a variety of different cells including glomerular and tubular epithelial cells, mesangial cells, fibroblasts as well as inflammatory cells as summarized by Li et al.^50^. Recent studies using single nuclei RNA sequencing in a kidney injury model demonstrated that C3 and C5 occurs primarily in renal tubular epithelial cells, while increased expression of complement receptors C3AR1 and C5AR1 occurs in interstitial cells including immune cells like monocytes/macrophages suggesting compartmentalization of complement components during kidney injury^51^. In our study, inflammatory cells seem to be an important source for at least complement factors C1q and C3. The increased complement expression is thus probably a result of the increased influx of these inflammatory cells, which, however, presumably influence the subsequent inflammatory reaction. The exact characterization of complement component expression profiles in rejected transplants needs detailed in future studies. In rejected transplants complement factors involved in classical and alternative pathway were highly expressed, while MASP2, as an activator of the lectin pathway, was expressed only on low levels. However, given the complexity of the situation it remains unclear which complement pathway is the key player in transplant pathology.

The main limitation of this study is that activation of complement takes place on the protein level, while the current study assesses expression on RNA level. This does not allow any direct conclusions about the activation of complement, as it is regulated by the activity of the various complement proteases. Furthermore, this study has a cross-sectional design and cannot differentiate between cause and consequence. In addition, the number of cases per group studied is relatively small and therefore the obtained results need to be validated in a larger cohort. Earlier studies demonstrated sex-dependent differences in serum complement factors of healthy volenteers^52^. In contrast, when we analyzed for the influence of donor sex, we could not observe any correlation of sex with the expression of complement genes. Recipient sex as a potential confounder in complement expression could not be analyzed in our study, since our control group consisted of males only. Finally, the exact role of complement in the pathophysiology of DGF and graft rejection can only be evaluated by inhibition of complement activation.

In conclusion, this study suggests an important role of complement in transplant pathology being triggered, at least in part, by CIT. Multiplex mRNA analysis might be a useful method to refine diagnosis and explore new pathways involved in rejection and delayed graft function in FFPE biopsies.

## Material and methods

### Renal tissue specimens

Kidney biopsies collected during and after renal transplantation between 2015 and 2020 from the Department of Nephrology at the FAU Erlangen-Nürnberg, Germany were included and did not contain tissues from prisoners. In total 28 FFPE carefully selected specimens of archived kidney biopsies (from the Department of Nephropathology, University Hospital, Erlangen, Germany) were used to evaluate the relevance of complement activation in patients with DGF (n = 6), ABMR (n = 7) and TCMR (n = 8) within the first 2.5 years. Biopsies from patients with borderline diagnosis or other co-morbidities like viral infection were excluded. Protocol biopsies from kidneys without signs of renal dysfunction or rejection, collected 12–14 months after renal transplantation, served as controls (n = 7). DGF was defined as impaired renal function necessitating dialysis within the first 10 days post transplantation and lack of rejection. The study groups and patients´ characteristics are described in Table 1 and BANFF scores are provided in details in a supplemental Table 1.

**Table 1:** Patient characteristics.

	Ctrl	DGF	TCMR	ABMR	Total
Recipient n = 29	7	6	8	7	28
Men (%)	7 (100%)	5 (71%)	2 (29%)	4 (57%)	18 (62%)
Age at transplant (years)	56 ± 12	51 ± 20	45 ± 12	49 ± 13	50 ± 15
Body mass index (kg/m^2^)	25 ± 5	26 ± 4	26 ± 4	24 ± 4	25 ± 4
Serum Creatinine (mg/dl)	1.24 ± 0.26	5.32 ± 2.67	2.95 ± 2.20	3.64 ± 1.79	3.22 ± 2.39
eGFR	69.0 ± 22.9	17.8 ± 16.2	37.4 ± 30.8	21.3 ± 13.5	37.0 ± 29.3
Smokers	3	3	0	1	7
Diabetes	2	2	0	2	6
Hypertension	7	6	4	6	23
CAD	1	3	1	1	5
Second transplantation	0	0	0	5	5
**Donor**					
Men (%)	2 (29%)	2 (33%)	4 (50%)	4 (57%)	12 (43%)
Age at transplant (years)	49 ± 19	55 ± 14	54 ± 7	55 ± 9	53 ± 12
Body mass index (kg/m^2^)	27 ± 4	28 ± 2	29 ± 8	29 ± 3	28 ± 5
Serum Creatinine (mg/dl)	1.19 ± 1.04	2.56 ± 2.37	1.36 ± 0.57	2.14 ± 2.31	1.74 ± 1.65
Smoker	0	1	2	2	5
Diabetes	0	1	0	1	2
Hypertension	1	3	2	4	10
CAD	1	2	1	1	5
Deceased	4	6	6	7	23
**Transplant conditions**					
CIT (min)	605 ± 313	611 ± 349	680 ± 299	800 ± 246	603 ± 338
WIT (min)	30 ± 8	46 ± 26	46 ± 20	32 ± 17	38 ± 19
ABO incompatibility	1	1	0	0	2
HLA mismatch (min–max)	4.7 (4–6)	4 (3–5)	3 (0–4)	2.9 (0–4)	3.6 (0–6)

Ctrl = control, DGF = delayed graft function, TCMR = T-cell mediated rejection, ABMR = antibody-mediated rejection, eGFR = estimated glomerular filtration ratio (according to CKD-EPI equation), CAD = coronary artery disease, CIT = cold ischemia time, WIT = warm ischemia time, HLA = human leucocyte antigen; Ranges are stated as mean ± SD.

### Ethical approval

The study has been approved by the Ethics Committee of the Friedrich-Alexander-University in compliance with the Helsinki Declaration (ethical approval number Re.-No.4415). The Ethics Committee waived the need for retrospective procurement of informed consent for the use of archived biopsy rest material in this fully anonymized study.

### Multiplex mRNA expression analysis by nanostring

For expression analysis, RNA was isolated with the RNAeasy FFPE Kit (Qiagen, Venlo, Netherlands) from 15 µm sections. Using the NanoDrop spectrophotometer (Thermo Fisher Scientific, Waltham, MA, USA) RNA concentration and purity were measured. Isolates with a 260/280 nm absorbance ratio below 1.4 were excluded. The samples with a mRNA concentration of 166–300 ng were dissolved in a volume of 25 µl H_2_O followed by concentration of the RNA using a Savant SPD111 SpeedVac (Thermo Fisher Scientific) at 35 °C for 24 min to a volume of 2–3 µl. Gene expression was quantified with the NanoString nCounter FLEX Analysis System (NanoString Technologies, Seattle, WA), including the three major steps of hybridization, preparation and analysis, according to manufacturer`s recommendations. The nCounter Human Organ Transplant Panel, containing 760 target genes and 10 internal reference genes was used^53^. According to the information provided by NanoString the primers are assumed to have a high specificity and do not detect other isoforms (such as MAp19 or sMAP for MASP2 gene, or Factor H-like protein 1 for CFH gene) or closely related proteins (such as Factor H-related proteins for Factor H), that is why we did not validate this ourselves. The panel contains 23 genes attributed to the complement system. In the following, the results of 17 of these genes were described in more detail, while 6 were not mentioned because their expression was almost identical to other genes (e.g. C1QA) or their expression level was below the background (e.g. MASP1, CRP). Quality control and normalization of the data were performed with the nSolver Analysis Software Version 4.0 using internal negative control probes, synthetic positive controls and selected housekeeping genes. Statistical evaluation was conducted with the GraphPad Prism Software Version 8.0 and further analysis with the nSolver Advanced Analysis Software Version 2.0.

### Multiplex immunohistochemistry

Here we performed 4 rounds of staining and stripping according to the study of Ehrenberg et al.^54^. Staining procedure was conducted on 2 µm sections of FFPE kidney biopsies. After blocking of endogenous peroxidase using 3% H_2_O_2_, antigen retrieval was conducted in target retrieval solution pH 6 (DAKO Deutschland, Hamburg, Germany) for 2.5 min in a pressure cooker. Sections were incubated over night at room temperature or 4 °C (ITGB2) with the primary antibodies. Following antibodies diluted in 50 mM Tris pH 7.4 were used: a mouse monoclonal antibody against human CD68 (M0876; Dako Deutschland); a mouse monoclonal antibody against human CD21 (M0784; Dako Deutschland); a rabbit monoclonal antibody against human CD4 (Ab133616; Abcam, Cambridge, UK) and a rabbit polyclonal antibody against human integrin beta-2 (ITGB2) (HPA016894; Sigma Aldrich). After washing with 50 mM Tris pH 7.4, sections were incubated with biotinylated secondary goat anti-rabbit IgG (BA-1000; Vector laboratories) or horse anti-mouse IgG (BA-2001, Vector laboratories). Bound antibodies were detected using ABC-Kit and Immpact-AMEC Red as a substrate (both from Vector laboratories). Finally, nuclei were counter-stained using hemalaun. Slides were covered with 70% glycerol and imaged on the slide-scanner Axio Scan.Z1 (Zeiss, Oberkochen, Germany).

For the stripping procedure, a BME/SDS puffer solution was prepared according to the protocol established by Gendusa et al.^55^: 10 ml of 20% SDS was mixed with 6.25 ml of 1 M Tris−HCl (pH 6.8), 83.75 ml of deionized (Millipore) water, and 0.8 ml of BME. Immunostained sections were incubated for 60 min in stripping solution preheated to 56 °C followed by four times washing in dH_2_O and storing in 50 mM Tris pH 7.4 supplemented with 0.05% Tween 20. After stripping, another staining round started with the next primary antibody. The whole staining and stripping procedure was repeated 3 times. Images of the scanned slides were evaluated with the software QuPath Version 0.2.3^56^. Thereby, the wand tool was used to recognize and localize the whole tissue area and with the pixel classifier the percentage of the positively (red) stained area was calculated. Threshold for background staining was set very high and was adjusted for every antibody.

### Injury scores

Histopathological changes were graded using the respective Banff classification score of 2013 and 2017 for renal transplant biopsies in the process of routine diagnosis^57,58^. Selected clinical parameters from the time point of biopsy collection were retrospectively investigated. In addition, transplantation relevant parameters like cold and warm ischemia time, recipient and donor data (age, BMI, serum creatinine) and renal inflammation were included for correlation analysis with the compartment-specific scores for all investigated complement factors.

### Statistics

All data were presented as scatter plots showing each single data point and means ± SD using bars and whiskers. Outliers were excluded according to the ROUT outlier test with Q = 1%. Only if outliers reduced numbers of included data sets, the changed numbers in group was mentioned in the figure or table legends; otherwise the whole dataset as described in 2.1 was used. After testing values for normal distribution using Kolmogorov–Smirnov test, data were analyzed using a one-way or two-way analysis of variance (ANOVA) followed by Tukey’s multiple comparison test or, if not normally distributed by Kruskal–Wallis test followed by Dunn’s multiple comparison test. Spearmans correlation analysis was performed to test for correlative relationships. *p* < 0.05 was accepted as statistically significant (*p < 0.05; **p < 0.01; ***p < 0.001; ****p < 0.0001).

## Supplementary Information

Supplementary Information.

## Data Availability

The data that support the findings of this study are available from the corresponding author upon reasonable request. Some data may not be made available because of privacy or ethical restrictions.

## Abbreviations

ABMR: Antibody-mediated rejection
BMI: Body mass index
CAD: Coronary artery disease
CIT: Cold ischemia time
Ctrl: Control
DGF: Delay graft function
FDC: Follicular dendritic cells
FFPE: Formalin-fixed paraffin-embedded
GFR: Glomerular filtration ratio
HLA: Human leucocyte antigen
IRI: Ischemia reperfusion injury
MAC: Membrane attack complex
PC: Principal component
TCMR: T-cell mediated rejection
WIT: Warm ischemia time
