# Supplementary material for: Multiplex gene analysis reveals T-cell and antibody-mediated rejection-specific upregulation of complement in renal transplants
Source: Sci Rep. 2021 Jul 29;11:15464. doi: 10.1038/s41598-021-94954-3 (PMC8322413; doi:10.1038/s41598-021-94954-3)
Supplement: Supplementary file 1 — Supplementary Information. [file 41598_2021_94954_MOESM1_ESM.docx]

**Multiplex gene analysis reveals T-cell and antibody-mediated rejection-specific upregulation of complement in renal transplants**

Eva Vonbrunn^1^, Tajana Ries^1^, Söllner Stefan^1^, Janina Müller-Deile^2^, Maike Büttner-Herold^1^, Kerstin Amann^1*^, Christoph Daniel^1*^

1. Department of Nephropathology
2. Department of Nephrology and Hypertension

Friedrich-Alexander-University (FAU) Erlangen-Nuernberg, Germany

*: these authors contributed equally to this work

Address of correspondence: Prof. Dr. Christoph Daniel

Department of Nephropathology

Krankenhausstr. 8-10

91054 Erlangen, Germany

Tel.:0049-9131-8522602

Email: Christoph.Daniel@uk-erlangen.de

**Supplemental Table 1: Banff classification**

|  | **Nr.** | **Baseline disease** | **Diagnostic findings** | **IFTA** | **g** | **i** | **ti** | **t** | **v** | **ptc** | **aah** | **cg** | **ci** | **ct** | **cv** | **mm** |
| --- | --- | --- | --- | --- | --- | --- | --- | --- | --- | --- | --- | --- | --- | --- | --- | --- |
| **Ctrl** | 1 | Diabetic Nephropathy | no evidence for rejection | 0 | 0 | 0 | 0 | 0 | 0 | 0 | 0 | 0 | 0 | 1 | 0 | 0 |
|  | 2 | IgAN | no evidence for rejection | 0 | 0 | 0 | 0 | 0 | 0 | 0 | 0 | 0 | 0 | 1 | 0 | 0 |
|  | 3 | Diabetic Nephropathy | no evidence for rejection | 0 | 0 | 0 | 0 | 0 | 0 | 0 | 0 | 0 | 0 | 0 | 0 | 0 |
|  | 4 | Unknown | no evidence for rejection | 1 | 0 | 1 | 1 | 0 | 0 | 0 | 0 | 0 | 1 | 1 | 0 | 0 |
|  | 5 | Nephrocalcinosis | no evidence for rejection | 1 | 0 | 0 | 1 | 0 | 0 | 0 | 2 | 0 | 1 | 1 | 3 | 0 |
|  | 6 | hypert. ischemic Nephropathy | no evidence for rejection | 1 | 0 | 0 | 0 | 0 | 0 | 0 | 0 | 0 | 1 | 1 | 0 | 0 |
|  | 7 | ADPKD | no evidence for rejection | 0 | 0 | 1 | 1 | 0 | 0 | 0 | 0 | 0 | 0 | 1 | 2 | 0 |
| **DGF** | 1 | FSGS | no evidence for rejection | 0 | 0 | 0 | 0 | 0 | 0 | 0 | 0 | 0 | 0 | 1 | 0 | 0 |
|  | 2 | FSGS, Reflux | no evidence for rejection | 0 | 0 | 0 | 0 | 0 | 0 | 0 | 1 | 0 | 0 | 0 | 1 | 0 |
|  | 3 | chronic interstitial Nephritis | no evidence for rejection | 1 | 0 | 0 | 0 | 0 | 0 | 0 | 1 | 0 | 1 | 1 | 1 | 1 |
|  | 4 | Unknown | no evidence for rejection | 0 | 0 | 0 | 0 | 0 | 0 | 0 | 1 | 0 | 0 | 0 | 0 | 1 |
|  | 5 | IgAN | no evidence for rejection | 0 | 0 | 1 | 1 | 0 | 0 | 0 | 2 | 0 | 0 | 1 | 0 | 0 |
|  | 6 | Cystinosis | no evidence for rejection | 0 | 0 | 1 | 1 | 0 | 0 | 0 | 0 | 0 | 0 | 1 | 0 | 0 |
| **TCMR** | 1 | Vesicoureteral Reflux | chronic active IB & acute IB | 3 | 0 | 3 | 3 | 3 | 0 | 1 | 2 | 0 | 3 | 3 | 3 | 3 |
|  | 2 | Bilateral shrunken kidney | acute IB | 2 | 0 | 2 | 2 | 3 | 0 | 0 | 2 | 0 | 2 | 2 | 1 | 0 |
|  | 3 | Unknown | acute IB | 2 | 0 | 3 | 3 | 3 | 0 | 1 | 2 | 0 | 2 | 2 | 3 | 0 |
|  | 4 | Granulomatosis w. Polyangiitis | acute IA | 1 | 0 | 2 | 2 | 2 | 0 | 0 | 2 | 1 | 1 | 1 | 2 | 1 |
|  | 5 | Hypertensive Nephropathy | acute IIA |  | 0 | 3 | 3 | 3 | 1 | 0 | 2 | 0 |  |  | 3 | 0 |
|  | 6 | Hypertensive Nephropathy | chronic active IB & acute IA | 3 | 0 | 2 | 2 | 2 | 0 | 0 | 2 | 0 | 3 | 3 | 1 | 0 |
|  | 7 | Vesicoureteral Reflux | acute IA | 1 | 0 | 2 | 2 | 2 | 0 | 0 | 1 | 0 | 1 | 1 | 0 | 0 |
|  | 8 | Benign Nephrosclerosis/ hypertensive Nephropathy | acute IIA | 2 | 0 | 2 | 2 | 3 | 0 | 0 | 1 |  |  |  |  |  |
| **ABMR** | 1 | Glomerulosclerosis | acute Type II | 0 | 1 | 1 | 1 | 1 | 0 | 1 | 0 | 0 | 0 | 1 | 0 | 0 |
|  | 2 | IgAN | acute Type II | 1 | 1 | 1 | 1 | 0 | 0 | 2 | 2 | 0 | 1 | 1 |  | 1 |
|  | 3 | aHUS | acute Type II | 0 | 0 | 0 | 0 | 0 | 0 | 2 | 0 | 0 | 0 | 1 | 0 | 0 |
|  | 4 | Diabetic-hypertensive NP | acute Type II | 1 | 1 | 1 | 1 | 1 | 0 | 1 | 0 | 0 | 1 | 1 | 1 | 0 |
|  | 5 | Nephrosclerosis | acute Type II | 0 | 0 | 0 | 0 | 0 | 0 | 2 | 0 | 0 | 0 | 1 | 0 | 0 |
|  | 6 | IgAN | acute Type II | 0 | 0 | 0 | 0 | 0 | 0 | 1 | 0 | 0 | 0 | 0 | 0 | 0 |
|  | 7 | ADPKD | chronic active & acute Type II | 1 | 2 | 1 | 2 | 1 | 0 | 1 |  | 3 | 1 | 1 | 2 | 2 |

**Supplemental table 2: Correlation of complement related gene expression with serum creatinine at biopsy and hyptertension of donor.**

| **Complement**  **related gene** | **Serum creatinine**  **at biopsy** | **Donor hypertension** |
| --- | --- | --- |
| **C1QA** | **0.422*** | **0.453*** |
| **C1QB** | **0.422*** | **0.492*** |
| **C1S** | 0.225 | 0.335 |
| **C3** | 0.169 | 0.138 |
| **C3AR1** | **0.497*** | **0.404*** |
| **C5** | **-0.525*** | -0.049 |
| **C5AR1** | **0.583**** | 0.305 |
| **C9** | 0.135 | 0.138 |
| **CD4** | 0,083 | 0.345 |
| **CD46** | **-0.485*** | -0.236 |
| **CD55** | **0.445*** | 0.256 |
| **CD59** | 0.135 | -0.217 |
| **CD68** | 0.376 | 0.335 |
| **CFB** | **0.516*** | 0.148 |
| **CFH** | 0.125 | 0.246 |
| **CR1** | 0.346 | **0.433*** |
| **ITGAM** | 0.347 | **0.384*** |
| **ITGAX** | 0.136 | **0.394*** |
| **ITGB2** | 0.179 | 0.286 |
| **MASP2** | 0.086 | 0.098 |
| **SERPING1** | **0.658**** | 0.089 |
